# Identification of immunogenic cell death-related subtypes used for predicting survival and immunotherapy of endometrial carcinoma through a bioinformatics analysis

**DOI:** 10.1097/MD.0000000000034571

**Published:** 2023-08-04

**Authors:** Zhen Liu, Yongjin Luo, Linhong Su, Xiaoxia Hu

**Affiliations:** a Jinan University, Guangzhou, China; b Department of Gynecology, The People’s Hospital of Guangxi Zhuang Autonomous Region, Guangxi Academy of Medical Sciences, Nanning, China.

**Keywords:** endometrial carcinoma, immune microenvironment, immunogenic cell death, immunotherapy, prognosis

## Abstract

Immunogenic cell death (ICD) is a unique phenomenon that can trigger comprehensive, adaptive immune responses through damage-associated molecular patterns, offering a promising avenue for tumor immunotherapy. However, the role of ICD-related genes and their correlation with endometrial carcinoma (EC), the most prevalent gynecologic malignancy, remains unclear. This study examined genetic, transcriptional, and clinical data of EC obtained from the Cancer Genome Atlas database. Unsupervised clustering analysis was utilized to identify distinct ICD clusters based on the expression of ICD-related genes. Regarding the different clusters, their survival analysis, assessment of the immune microenvironment, immune cell infiltration, immune checkpoint analysis, and tumor mutation burden analysis were performed. Furthermore, an ICD risk signature was established using univariate Cox regression and least absolute shrinkage and selection operator analysis. The Chi-square test was employed to investigate the relationship between the ICD score and clinical features. Multiple computational analytical tools were used to assess immune annotation, somatic mutations, tumor mutation burden, and response to immunotherapy and chemotherapy drugs in different ICD score groups. Two ICD clusters were identified, indicating that the ICD-high cluster was associated with improved prognosis, abundant immune cell infiltration, and enrichment of pathways related to immunologic activation. Moreover, the ICD risk signature showed predictive value for the immune microenvironment, immunotherapy response, chemotherapy susceptibility, and prognosis in EC. Our findings offer novel insights into personalized treatment strategies for EC patients.

## 1. Introduction

An estimated 417,000 new cases of endometrial cancer (EC) were reported worldwide in 2020, which is the sixth most common cancer in women.^[1]^ Endometrial hyperplasia serves as a precursor to EC. Recent reports suggest that the activity of the TRPM7 ion channel may indicate the progression from endometrial hyperplasia to early-stage EC.^[2]^ While early-stage EC cases can often be successfully treated through a hysterectomy, advanced EC poses significant challenges with a bleak prognosis and a lack of reliable markers and effective treatment options. However, advancements in targeted chemotherapeutic approaches, such as immunotherapy targeting immune checkpoints and novel chemotherapy strategies, have emerged based on the molecular understanding of EC.^[3]^ Therefore, further exploration of molecular mechanisms through ongoing studies will enhance treatment strategies for advanced EC and improve the prognosis prediction for EC patients.

Immunogenic cell death (ICD) is a type of regulated cell death triggered by chemotherapeutic agents, radiotherapy, oncolytic viruses, physicochemical therapies, and photodynamic therapy.^[3,4]^ ICD can activate immunogenic signals called damage-associated molecular patterns (DAMPs) in dead tumor cells.^[5]^ DAMPs include cytokines (such as type I interferons [IFNs] and interlukin-1 family, the cell surface exposure of “eat me” signals and heat-shock proteins (HSP70 and HSP90), nucleic acids pathway, high mobility group box1, and extracellular release of immunostimulatory factors, such as adenosine triphosphate.^[4,6–8]^ ICD and its association with DSMPs may reportedly improve the outcomes of various cancers. For example, during ICD, rapid production of type I IFN is stimulated by malignant cells via activation of the endosomal pattern recognition toll-like receptor 3 (TLR3). On the other hand, secreted IFN can bind to IFN-α and IFN-β receptors expressed on the surface of cancer cells to trigger the release of chemokine (C-X-C motif) ligand 10, which mediates chemotaxis and immune stimulation to cancer cells.^[9]^ As mentioned above, the ICD process of DAMPs induces the secretion of immunogenic signals in tumor cells, which can also activate dendritic cells to attenuate immunosuppression in tumors.^[10]^ Previous studies showed that some chemotherapeutic agents, such as cyclophosphamide, bleomycin, doxorubicin, epirubicin, mitoxantrone, idarubicin, and bortezomib could induce ICD and stimulate the anti-tumor immune response.^[11]^ However, the role of ICD-related genes and their correlations with EC remains unclear.

This study analyzed the genetic, transcriptional, and clinical data of EC collected from the Cancer Genome Atlas (TCGA) database. This study systematically examined the correlation between genes related to immune cell dysfunction (ICD) and the clinicopathological features of patients with EC. As a result, 2 ICD clusters were identified, along with an ICD risk signature. Utilizing these ICD clusters and the ICD-related risk signature, we identified biomarkers associated with ICD, enabling the prediction of the immune microenvironment status, chemotherapy susceptibility, and prognosis of EC patients. The results of this study provide new insights into individualized treatment strategies for EC patients.

## 2. Materials and methods

### 2.1. Datasets and preprocessing

The RNA sequence data and the interrelated clinical information of 541 EC samples, such as therapeutic information, somatic mutation data, copy number variation data, and information about 23 normal human endometrial samples, were collected from the TCGA (https://portal.gdc.cancer.gov/). Those data were randomly separated into the training or test group at a ratio of 5:5. A total of 34 immune cell dysfunction-related genes (ICDRGs) were identified by the previous studies (Table 1). Statistics were performed based on clinical variables, such as tumor stage, grade, and patient age. The R package of “limma” was used to identify differentially expressed genes in the tumor tissues listed in the TCGA database, according to a cutoff *P* value of .05. The intersection of differentially expressed genes and ICDRGs was displayed using the “pheatmap” R package (4.1). In this study, all information and clinical matrix involved were downloaded from the public database. Approval from the ethics committee and written informed consent from patients were not required.

**Table 1:** Immunogenic cell death-related genes.

Gene symbol	Description	Gene symbol	Description	Gene symbol	Description
ATG5	Autophagy related 5	IL10	Interleukin 10	CASP1	Caspase1
CD4	Cluster of differentiation 4	CASP8	Caspase8	CD8A	Cluster of differentiation 8A
BAX	BCL2-Associated X	CD8B	Cluster of differentiation 8 beta	CXCR3	C-X-C Motif Chemokine Receptor 3
CALR	Calreticulin	TLR4	Toll-like receptor 4	LY96	Lymphocyte Antigen 96
P2RX7	Purinergic receptor P2X 7	NT5E	Ecto-5’-nucleotidase	EIF2AK3	Eukaryotic Translation Initiation Factor 2 Alpha Kinase 3
IL1R1	Interleukin 1 receptor type 1	IL17A	Interleukin 17A	PDIA3	Protein Disulfide Isomerase Family A Member 3
TNF	Tumor necrosis factor	PIK3CA	Phosphatidylinositol-4,5-Bisphosphate 3-Kinase Catalytic Subunit Alpha	IL6	Interleukin 6
NLRP3	NOD-, LRR-, and pyrin domain-containing protein 3	MYD88	Myeloid differentiation primary response 88	IFNGR1	Interferon gamma receptor 1
IL1B	Interleukin 1 beta	IL17RA	Interleukin-17 receptor A	IFNA1	Interferon Alpha 1
IFNG	Interferon gamma	IFNB1	Interferon beta 1	FOXP3	Forkhead Box P3
HSP90AA1	Heat Shock Protein 90 Alpha Family Class A Member 1	HMGB1	High mobility group box1	PRF1	Perforin 1
ENTPD1	Ectonucleoside triphosphate diphosphohydrolase 1				

### 2.2. Data analysis of ICDRGs using consensus clustering

Unsupervised clustering analysis was used to identify the different ICD patterns based on the expression of ICDRGs. The identified ICD patterns were used for further analysis of 541 tumor samples. The R package of “ConsensClusterPlus” was used for the above analysis, which was repeated 1000 times to ensure cluster stability. The Kaplan–Meier survival curve was used to estimate the overall survival rate.

### 2.3. Immune infiltration landscape analysis

The proportions of 22 immunocytes infiltrating the tumor were measured using the CIBERSORT methodology, the packages of “e1071” and “preprocessCore” in R, and the LM22 signature retrieved from the CIBERSORT web portal. The biological processes were investigated by gene set variation analysis enrichment analysis. The immune, stromal, and ESTIMATE scores and tumor purity were estimated by the “ESTIMATE” package. Spearman’s correlation analysis was used to verify the differential expression of the immune checkpoint molecules. The Kruskal test was used to compare the differences in the immune subtypes and the expression of HLA-related genes between different groups.

### 2.4. Establishing a prognosis signature related to ICD genes

A unitive signature was developed to predict overall survival based on clinically significant characteristics and the ICDRGs in EC. First, the univariate Cox regression analysis of all possible ICDRGs was performed to identify the prognostic genes with a significant prognostic value (*P* < .05) in the training set. Further analysis using the least absolute shrinkage and selection operator Cox regression model and the R package of “clusterSur” narrowed the range of candidate genes, followed by the multivariate Cox analysis to establish a signature. The TCGA-listed samples (n = 541) were divided into a training group (n = 272) and a test group (n = 269), with the propensity score matching model. According to the median risk scores, all samples were also divided into low-risk (ICD score < median) and high-risk (ICD score > median) groups. The Kaplan–Meier survival curve was performed to compare the difference in survival outcomes between the 2 groups. The R packages of “risk plot” and “ROC” were used for risk factor analysis and receiver operating characteristic curve analysis, respectively. Finally, the survival rates of both high-risk and low-risk groups were also evaluated. In addition, time-dependent ROC curves for 1-, 3- and 5-year survival were used to assess the ability of the signature to predict the prognosis of the EC patients in both high-risk and low-risk groups.

### 2.5. Independence of the signature risk score and its clinical relationship with other clinicopathological features

The chi-square test was used to investigate the relationships between ICD score and clinical features (age, grade, and tumor stage). The univariate and multivariate analyses were performed to determine if the risk score could be used as an independent prognostic factor, that is, independent of other clinical and pathological factors. A stratum analysis was also performed to determine whether the ICD score retained its predictive power in the different subgroups.

### 2.6. Immune status analysis

To reflect the immune status of the analyzed samples, the difference in several scores between the high- and low-risk groups was graphically displayed as a violin chart by the ESTIMATE algorithm, including the immune status score, microsatellite instability score, dysfunction score, exclusion score, tumor immune dysfunction score, and exclusion total score. The R package of “immunocor” was used to quantify the infiltration of immune cells in both high- and low-risk groups. Several programs, such as TIMER (https://cistrome.shinyapps.io/timer/), CIBERSORT (https://cibersortx.stanford.edu/. stanford.edu/), CIBERSORT-ABS, QUANTISEQ, MCPcounter, EPIC, and XCELL (https://xcell.ucsf.edu/) were used to estimate the proportion of tumor-infiltrating immune cells in the high- and low-risk groups. Single-sample gene set enrichment analysis (ssGSEA), a variation of the gene set enrichment analysis (GSEA) algorithm, was used to evaluate the association of 28 infiltrating immune cell types in the high- and low-risk groups. Spearman’s correlation analysis was used to evaluate the differential expression of the immune-checkpoint molecules between the high- and low-risk groups. The MHC genes that belong to the immunoinhibitory and immunostimulatory genes are involved in the tumor evasion mechanisms. The analysis data of MHC genes were obtained from the previous study.^[12]^ Calculation of immunophenoscore (IPS) was performed using the Cancer Immunome Database (https://tcia.at/home).^[13]^ Based on the status of cytotoxic T lymphocyte antigen 4 (CTLA-4) and programmed death-1 (PD-1) expression, the difference in IPS between the high- and low-risk groups was compared.

### 2.7. Gene mutation analysis

Based on the somatic mutation data from TCGA, re-analysis of the gene mutation in the EC patients was performed using the R package of “maftools,” thereby the tumor mutation burden (TMB) of each patient was calculated, and the comparison of the difference of TMB between the high- and low-risk groups was implemented. Survival analysis was also completed according to the TMB score of the EC patients.

### 2.8. Chemotherapy response analysis

The chemotherapy response of the EC patients to chemotherapeutic agents was estimated using the Genomics of Drug Sensitivity in Cancer database. The half maximal inhibitory concentration (IC50) of anticancer drugs in the high- and low-risk groups was determined by the R package of “oncoPredict.”

### 2.9. Statistical analysis

R survival package was used for survival analysis, and the survival rate of each group was tested by the Log-Rank test hypothesis. The Kruskal–Wallis test was used to compare the difference in data of two or more groups. The Wilcoxon test was also used to compare the difference in data between the 2 groups. The Kaplan–Meier method was used to generate the survival curve of each subgroup in the data set. The chi-square test was used to compare the difference in the mutation frequency between the ICD score subgroup and somatic cells. Spearman correlation analysis was used to calculate the correlation coefficient. All statistical analyses were performed using R versions 4.1.0 and 4.0.0. The statistical significance was set to *P* < .05.

## 3. Results

### 3.1. Expression levels and copy number of ICDRGs in EC

**Figure 1.**
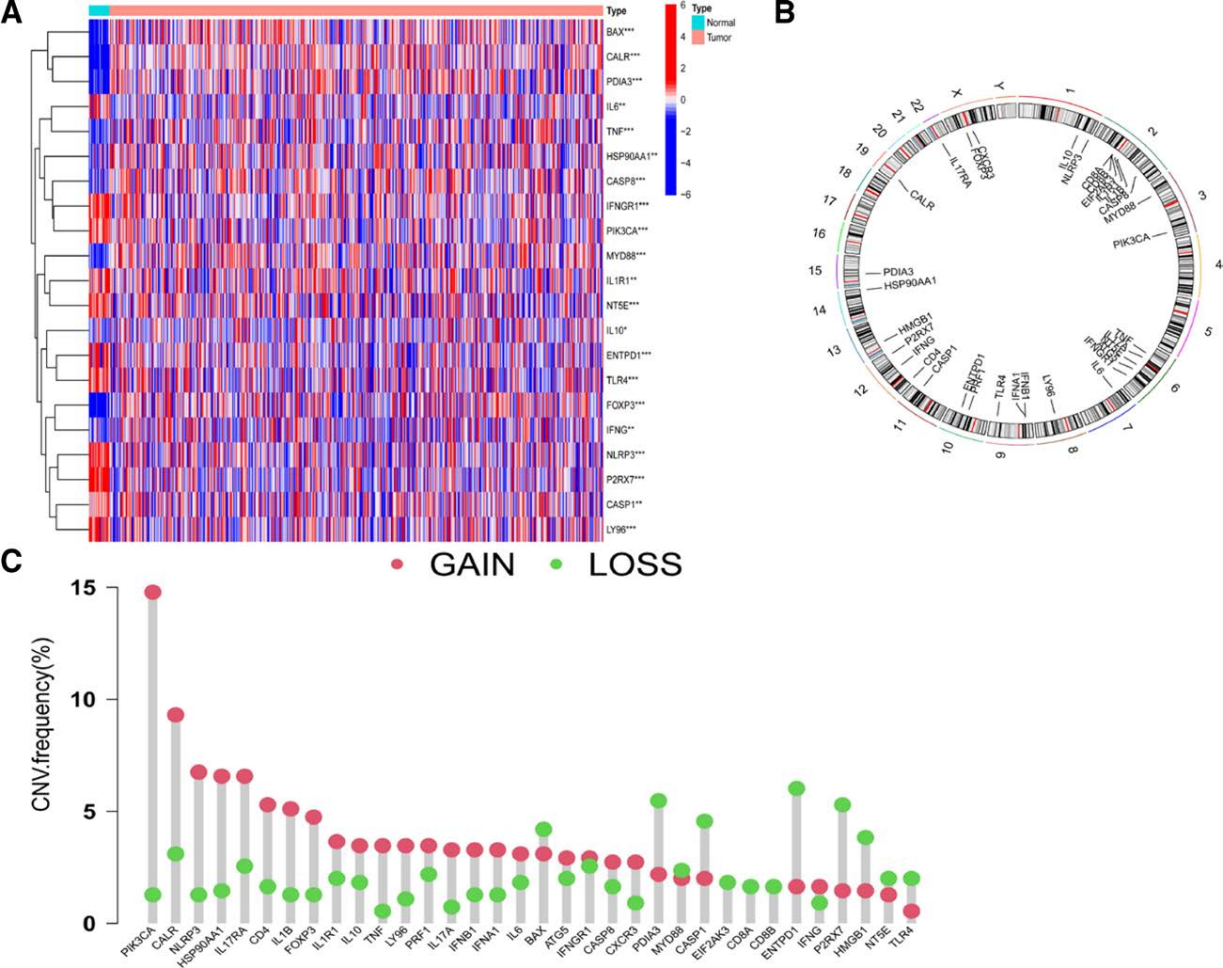
Differential expression and variations of 34 ICDRGs in endometrial cancer. (A) There were 11 upregulated ICDRGs and 10 downregulated ICDRGs in endometrial cancer samples. (B) The sites of CNV in CRGs on the 23 chromosomes. (C) The distributions of CNV gains and losses among ICDRGs. CNV = copy-number variation, CRGs = cuprotosis-related genes, ICDRGs = ICD-related genes.

A total of 11 downregulated ICDRGs and 10 upregulated ICDRGs were found in the samples of EC (Fig. 1A). Based on a review of the variations in the copy number, most ICDRGs were accumulated if the copy number was lost or deleted. All 34 ICDRGs underwent a change in frequent copy number (Fig. 1B and C).

### 3.2. Samples were stratified into 2 clusters based on the expression of ICD genes

**Figure 2.**
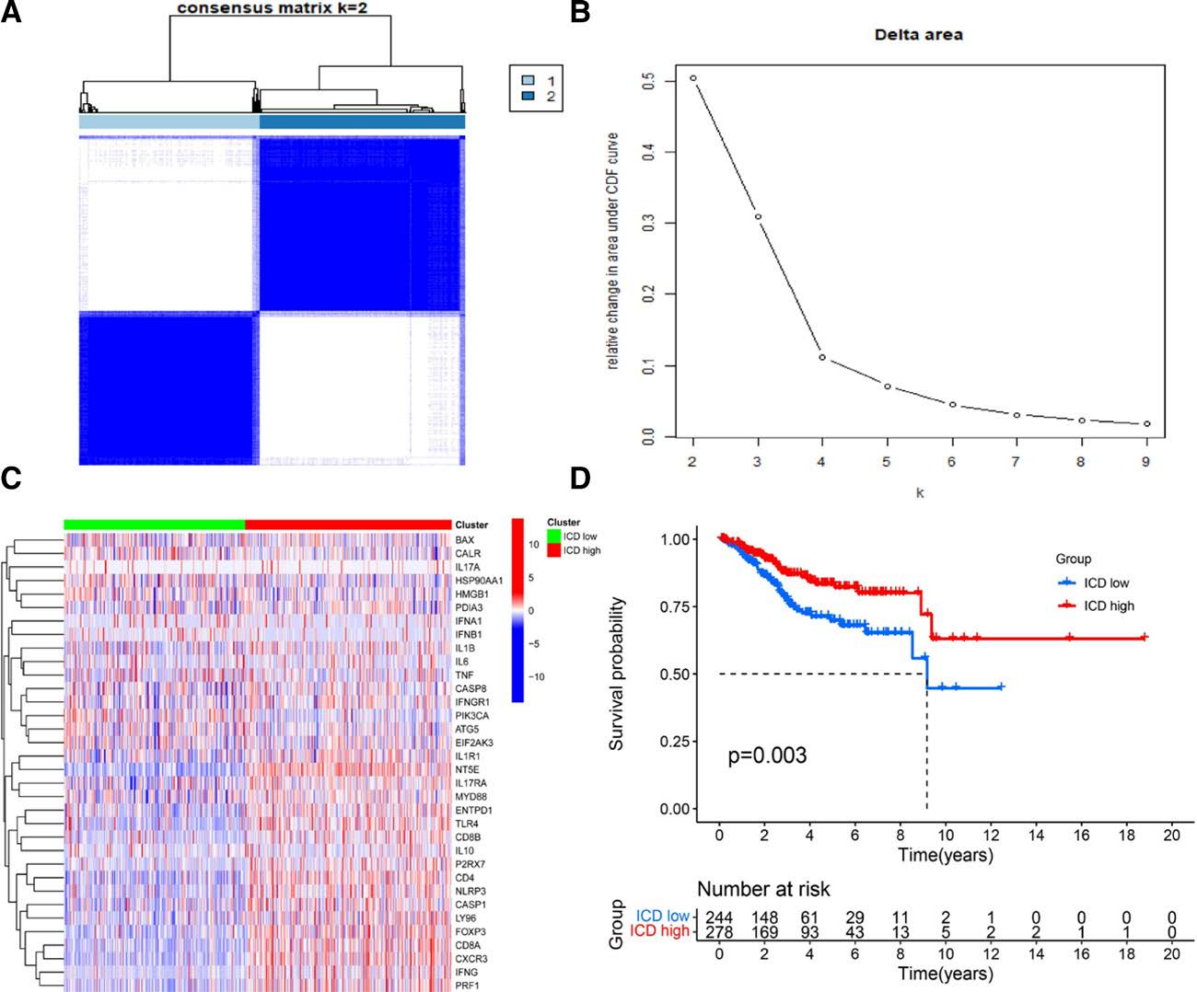
Identification of 2 clusters based on the expression of ICD genes. (A) The consensus matrix’s heatmap of 2 clusters (κ = 2). (B) Relative change in area under CDF curve. (C) The heatmap of ICDRGs distribution between those 2 clusters. (D) Subtype-specific Kaplan–Meier OS curves. CDF = cumulative distribution function, ICD = immunogenic cell death, ICDRGs = ICD-related genes, OS = overall survival.

The consensus cluster analysis in 541 EC samples found a cluster of κ = 2, with a difference between the highest intragroup and lowest intergroup among the cohort. This cluster was further divided into 2 groups: cluster A (ICD low, n = 243) and cluster B (ICD high, n = 78), based on the expression levels of ICDRGs (Fig. 2A and B). A heatmap was prepared based on the expression patterns of ICDRGs in those 2 clusters. Figure 2C shows that ICDRGs were more abundant in cluster B with a better prognosis than cluster A, based on the Kaplan–Meier survival curves of those 2 clusters (Fig. 2D).

### 3.3. Functional enrichment analysis and tumor microenvironment (TME) infiltration in 2 ICD clusters

**Figure 3.**
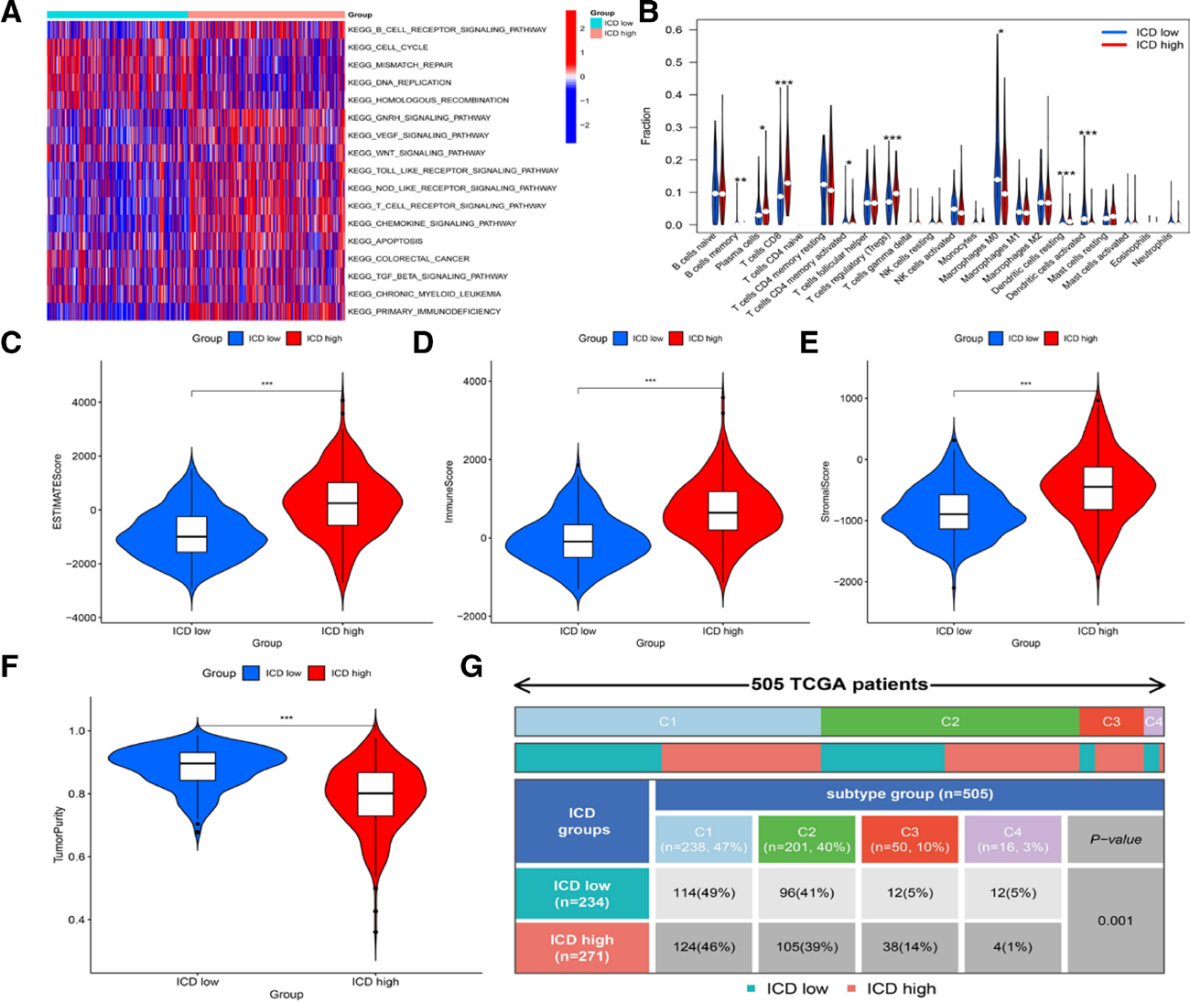
The correlation of the subtypes of ICD with TME invasion (A) ICD subtype-related cellular pathways as indicated by GSVA. (B) Correlations of the infiltration levels of tumor immune cells with 2 ICD subtypes. (C–F) Levels of the immune score, stromal score, estimate score, and tumor purity score in 2 ICD clusters. (G) Expression levels of ICDRGs in different immune subtypes. GSVA = gene set variation analysis, ICD = immunogenic cell death, ICDRGs = ICD-related genes, TME = tumor microenvironment.

GSVA, a particular type of gene set enrichment method, was used to examine the potential effects of both clusters on biological behavior. Compared to cluster A, cluster B showed enrichment in the pathways associated with immunologic activation. Cluster B had a remarkable enrichment in the immune-related pathways, such as the T cell receptor signaling pathway, B cell receptor signaling pathway, primary immunodeficiency, and chemokine signaling pathway (Fig. 3A). The relationship between ICD clusters and tumor immune cells was investigated by CIBERSORT, a versatile computational method for quantifying cell fractions from bulk tissue gene expression profiles. The results showed that the cluster ICD high had higher infiltration levels of the immune cells, such as CD8 T cell and T cells regulatory (Fig. 3B). Calculation of multiple functional scores, including stromal score, tumor purity score, estimate score, and immune score, was performed by the ESTIMATE algorithm. Compared to the ICD-low group, the levels of the immune score, stromal score, and estimated score were higher, but the tumor purity score was lower in ICD-high group (Fig. 3C–F). After the gene expression analysis using 505 EC samples, the expression levels of ICDRGs showed variations in multiple immune subtypes, including those related to wound healing (C1), IFN-gamma dominant (C2), inflammatory (C3), lymphocyte-depleted (C4), immunological quiet (C5), and TGF-beta dominant (C6), as shown in Figure 3G.

### 3.4. Somatic mutation distribution and expression of immune checkpoint-related genes in 2 ICD subtypes

**Figure 4.**
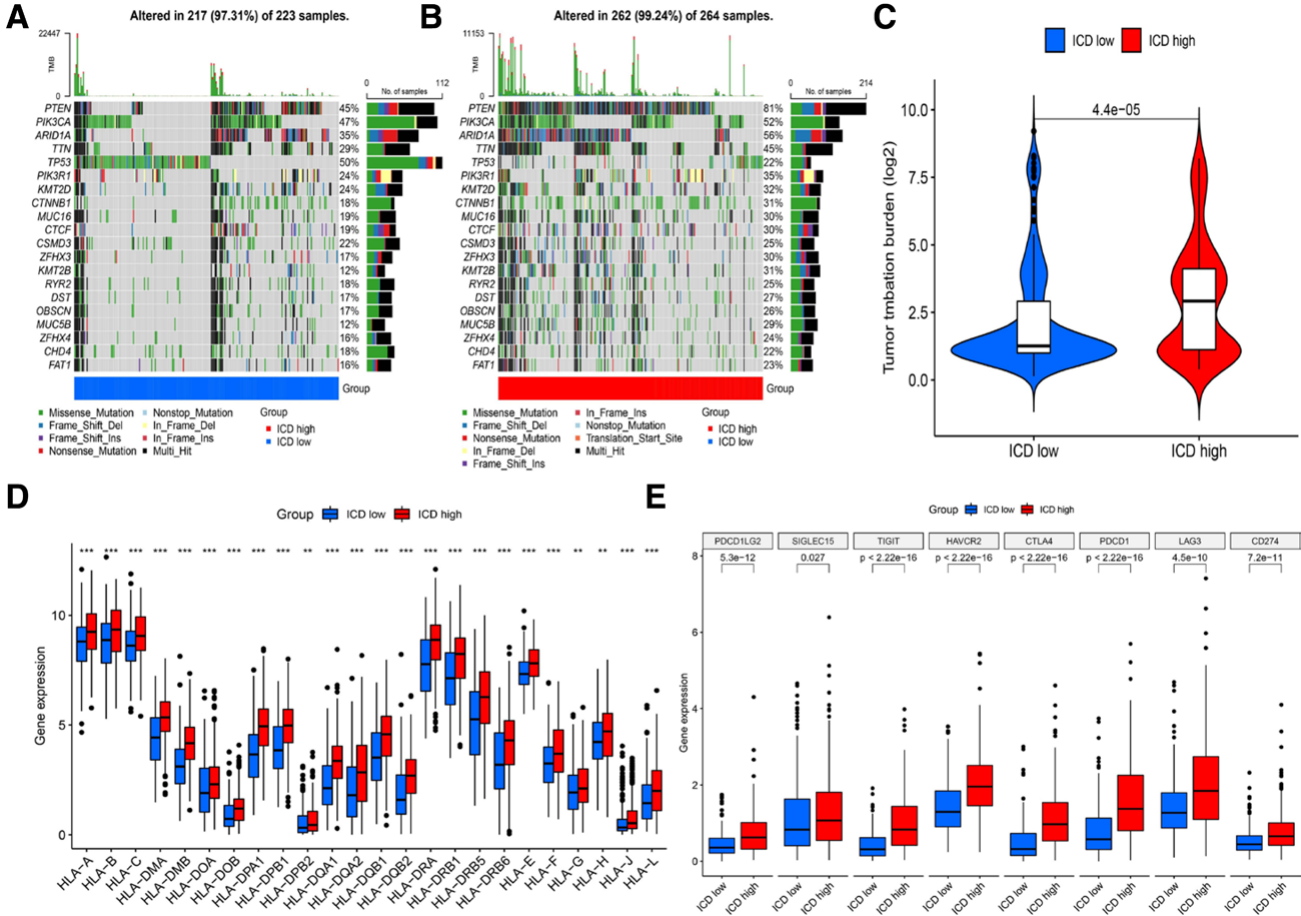
Comparison of the differences in somatic mutations and expression of immune checkpoint-related genes between 2 ICD clusters. Top 10 of the most frequently mutated genes in the ICD-high subtype (A) and ICD-low subtype (B) (visualized by Oncoprint). (C) The levels of TMB in 2 ICD clusters. (D) The expression levels of the different HLA-related genes in 2 ICD clusters. (E) The expression levels of the immune checkpoint-related genes in 2 ICD clusters. ICD = immunogenic cell death, HLA = human leukocyte antigen.

The differences in somatic mutation distribution between the 2 ICD clusters were examined by the maftools software package, indicating that the somatic mutation landscape was similar in cluster ICD high (99.24%) and cluster ICD low (97.23%). However, the mutational probability of PTEN in the cluster ICD-high group (81%) was higher than in the cluster ICD-low group (45%) (Fig. 4A and B). The EC patients in the cluster ICD-high group had a higher level of TMB than in the cluster ICD-low group (Fig. 4C). Moreover, the expression of most of the HLA-related genes was upregulated in the cluster-high ICD group (Fig. 4D). As shown in Figure 4E, the expression of the immune checkpoint-related genes, such as PDCD1 (PD-1), CD274 (PD-L1), and CTLA4, which are potential immunotherapy targets, were significantly upregulated in the cluster ICD-high group.

### 3.5. Construction of the ICD risk signature

**Figure 5.**
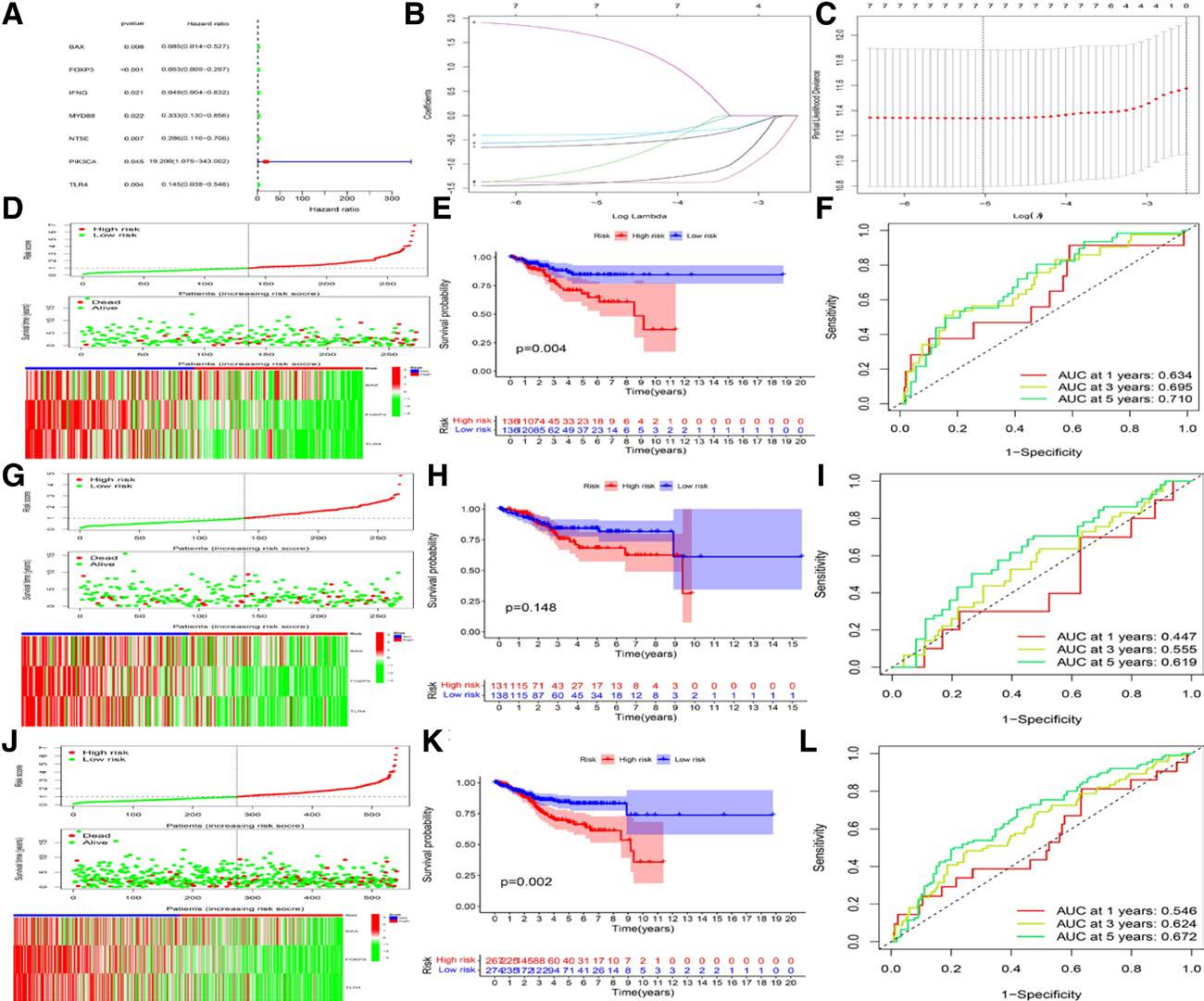
Construction of the ICD risk signature and comparison of the differences between high- and low-risk groups. (A) Evaluation of the prognostic value of the ICD genes in terms of OS by univariate Cox analysis. (B and C) Identification of 7 genes closely associated with OS by LASSO Cox analysis. (D, G, and J). The survival status, risk scores distribution, and the signature of 3 prognostic genes in different sets, including (D) training set, (G) test set, and (J) TCGA set. (E, H, and K) The prognostic significance of the risk model in different sets. (F, I, and L) AUC values in different sets, including (F) training set, (I) test set, and (L) TCGA set. AUC = area under curve, ICD = immunogenic cell death, LASSO = least absolute shrinkage and selection operator, OS = overall survival, TCGA = the Cancer Genome Atlas.

A unitive signature was established to predict the overall survival of the EC patients, according to the clinically significant characteristics and the expression levels of ICDRGs in EC samples. In the univariate Cox regression and least absolute shrinkage and selection operator analysis, 7 candidate genes had *P* value < .05 (Fig. 5A–C). After narrowing the range of candidate genes by multivariate Cox, 3 genes (including BAX, FOXP3, and TLR4) were selected for calculating the risk score using the formula “Risk score = (−2.035*BAX) + (−2.120*FOXP3) + (−1.179*TLR4)” in the multivariate Cox analysis. Three cohorts of the EC patients, including the training set (Set A, n = 272), test set (Set B, n = 269), and all TCGA set (Set C, n = 541), were enrolled to compare the differences in survival status, risk score distribution, survival probability, and related gene expression between 2 ICD subtypes. Results showed that the survival rate in the low-risk group was significantly higher than that in the high-risk group in 3 cohorts (Fig. 5D, G, and J). In the high-risk subgroup of either training set or TCGA set, the prognosis of the EC patients was poorer, compared to those in the low-risk subgroup (Fig. 5E, H, and K). The performance of the predictive model was evaluated based on the time-dependent ROC curve and area under curve values at different time points. As shown in Figure 5F, I, and L, the values of area under curve in Set A, Set B, and Set C were 0.634, 0.447, and 0.546 (at 1 year); 0.695, 0.555, and 0.624 (at 3 years); and 0.710, 0.619, and 0.672 (at 5 years), respectively.

### 3.6. ICD score as a prognostic biomarker in EC

**Figure 6.**
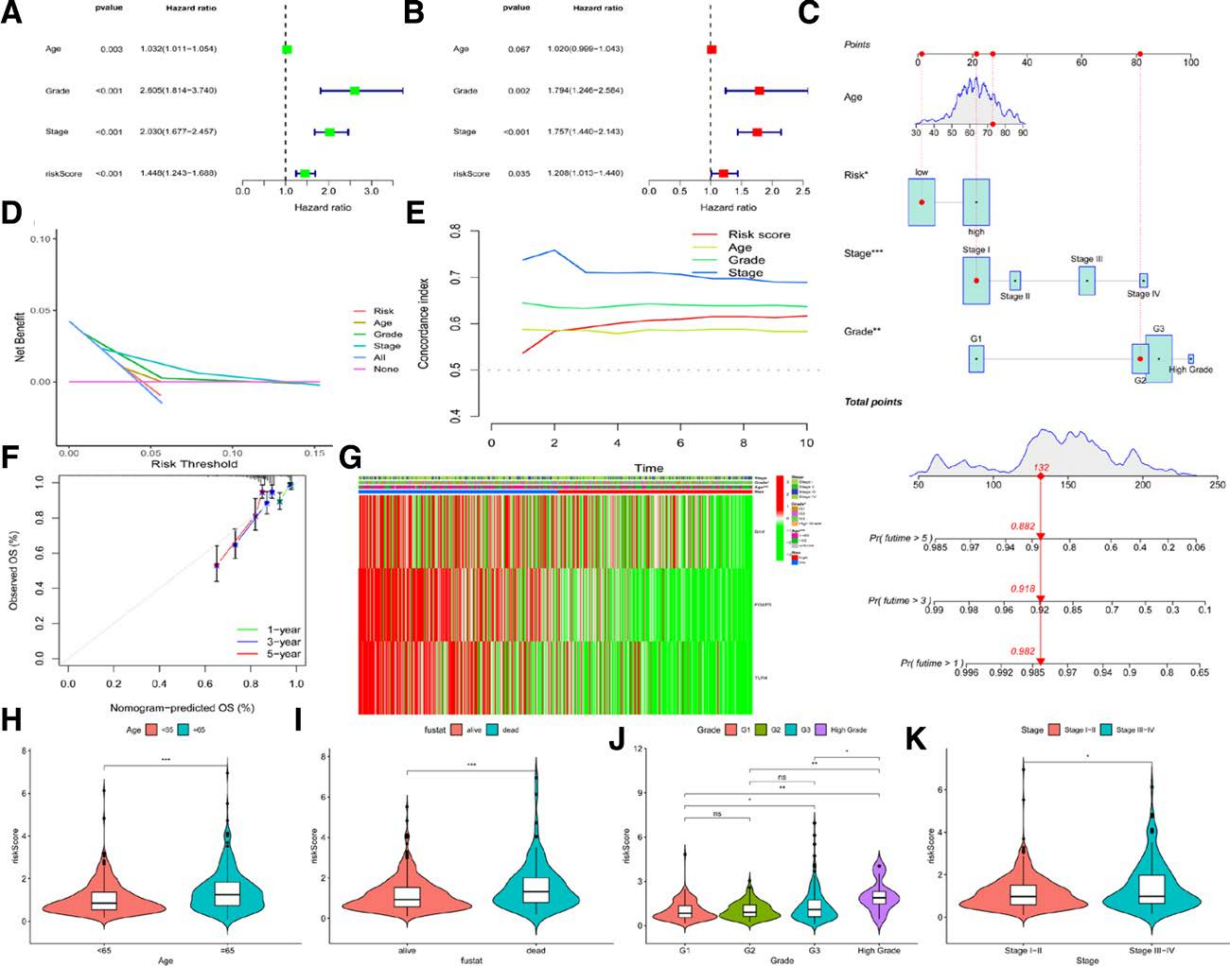
ICD score as a prognostic biomarker in EC. (A and B) Assessment of the value of ICD risk signature as an independent prognostic factor in EC by the univariate and multivariate Cox analyses. (C) Prediction of the 1-, 3-, and 5-years OS of the EC patients by a nomogram model established using their risk score and clinicopathological characteristics. (D–F) Difference in the predictive capacity of ICD risk signature between different groups and actual OS rate of 1-, 3-, and 5-years. (G) Differences in the clinicopathological traits and the expression levels of ICDRGs across subtypes. (H–K) A significant association of the risk score with tumor stage, clinical grade, and patients’ age. EC = endometrial carcinoma, ICD = immunogenic cell death, ICDRGs = ICD-related genes, OS = overall survival.

The value of ICD risk signature as an independent prognostic factor in EC was evaluated by the univariate and multivariate Cox analyses, as shown in Figure 6A and B. Additionally, to predict the overall survival (OS) rate of 1, 3, and 5 years of EC patients, their ICD-related prognostic signature and clinicopathological features were used to develop a nomogram model (Fig. 6C–F). The relationship between the features of clinical significance and ICDRGs across subtypes was displayed through a heatmap (Fig. 6G). Moreover, our study also revealed a significant association of the risk score with tumor stage, clinical grade, and age (Fig. 6H–K).

### 3.7. Immunization annotation in the different ICD score groups

**Figure 7.**
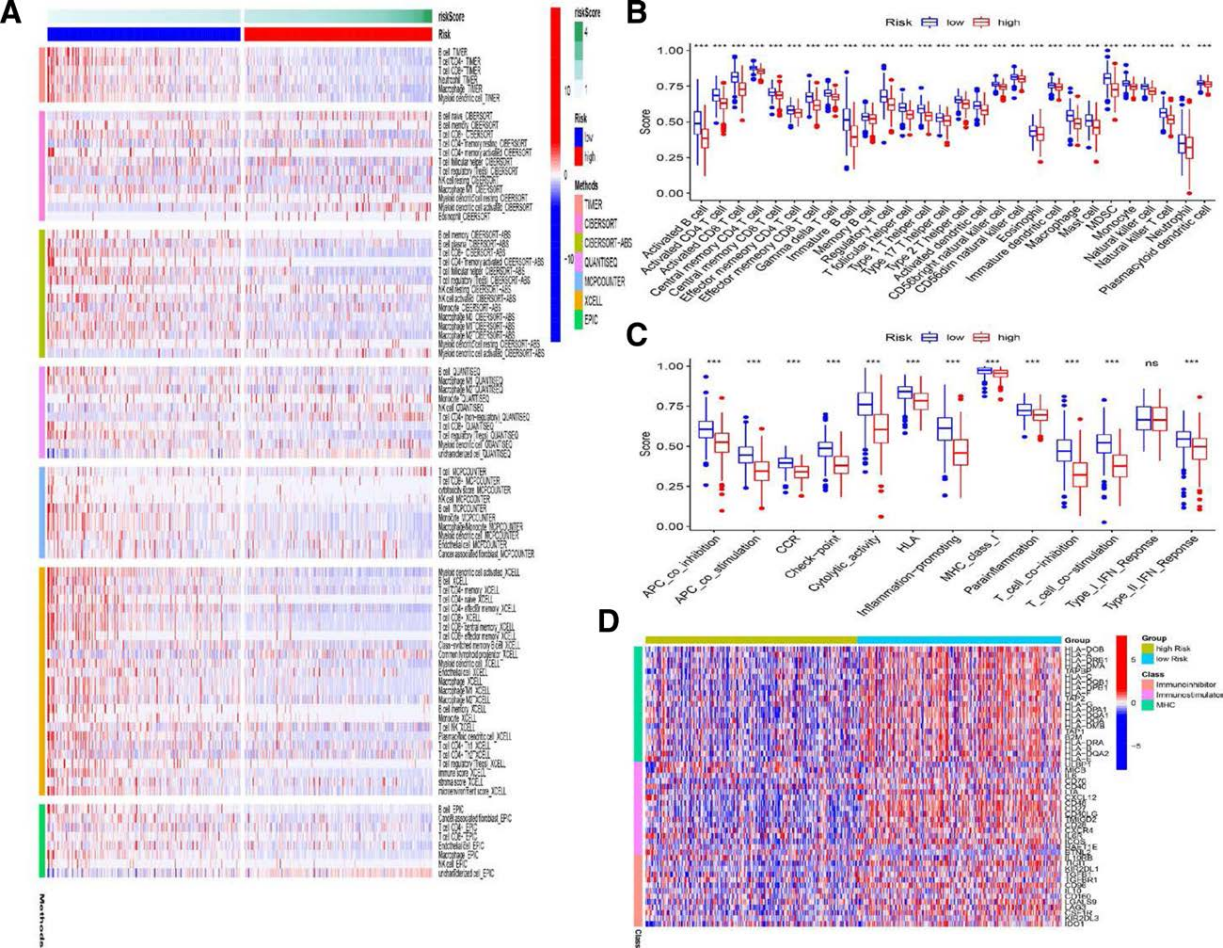
Immunization annotation in the different ICD score groups. (A) Enrichment analysis of multiple types of immune cells in low-risk and high-risk groups. (B and C) The fraction of 28 types of immune cells and their immune functions in the 2 groups as mentioned above. (D) The immunophenotypes were different between the 2 subgroups. ICD = immunogenic cell death.

The composition of immune cells was analyzed using multiple computational analytical tools, including CIBERSORT, TIMER, QUANTISEQ, CIBERSORT-ABS, EPIC, XCELL, and MCPcounter. The analysis results were visualized by a heatmap (Fig. 7A), which showed the significant difference in the composition of tumor-infiltrating immune cells, such as CD8+T cells, memory CD4+T cells, B cells, and cancer-associated fibroblast between the high- and low-risk groups. Enrichment analysis of the immune cells and their immune functions were also performed using ssGSEA, which showed a significant difference in the distribution of immune cells between the 2 groups. As shown in Figure 7B, almost all the immune cells were enriched in the low-risk group. ssGSEA data also showed that almost all the immune functions (except for type I IFN response) were significantly enriched in the low-risk group (Fig. 7C). Upregulation of expression of the immune-related genes, such as immunoinhibitory and immunostimulatory genes, and the genes of the MHC family was found in the low-risk group (Fig. 7D).

### 3.8. Analysis of somatic mutations and TMB in 2 ICD subtypes

**Figure 8.**
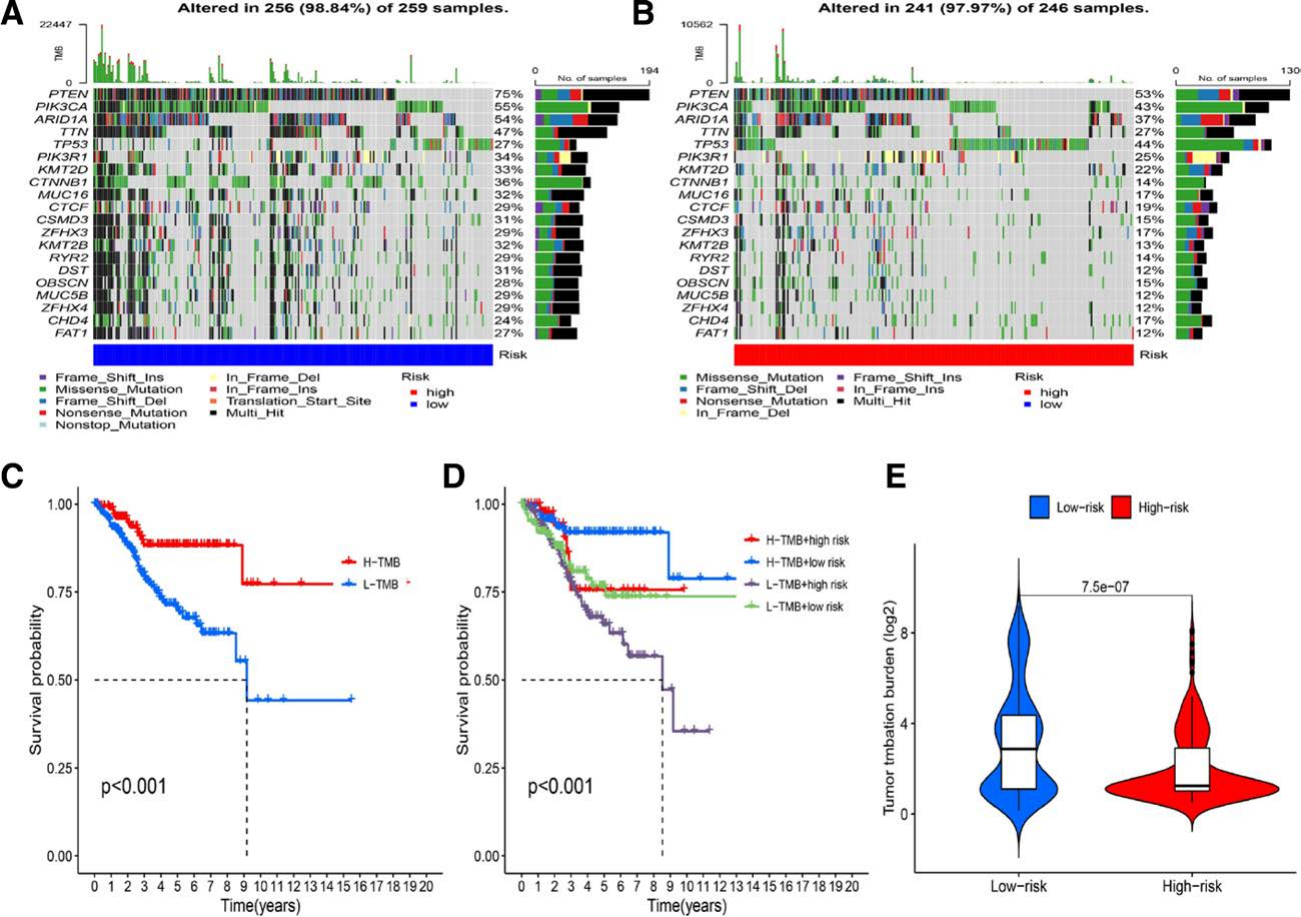
Comparison of somatic mutation differences between 2 ICD score subgroups. (A and B) Top 20 most frequently mutated genes in ICD high-risk group (A) and ICD-low risk group (B) revealed by Oncoprint program; (C) survival of the EC patients, according to their TMB score; (D) survival of the EC patients, according to their TMB score and ICD score; (E) TMB levels in the 2 ICD subtypes. EC = endometrial carcinoma, ICD = immunogenic cell death, ICDRGs = ICD-related genes, TMB = tumor mutation burden.

The difference in the distribution of somatic mutations between the groups of low and high ICD scores was evaluated by the maftools package. As shown in Figure 8A and B, the mutation rate between the low (98.84%) and high ICD score (97.97%) groups was the same. The TMB level in the high-risk group was significantly lower than in the low-risk group (Fig. 8E, *P* < .001). Moreover, the survival of the EC patients in the high-TMB group was better than that in the low-TMB group (Fig. 8C). Results also showed the best survival rate in either the low ICD score or the high-TMB group. In contrast, the survival rate was lowest in both the high ICD score and low-TMB groups (Fig. 8D).

### 3.9. Expression of the immunotherapy response-related genes in 2 ICD subtypes

**Figure 9.**
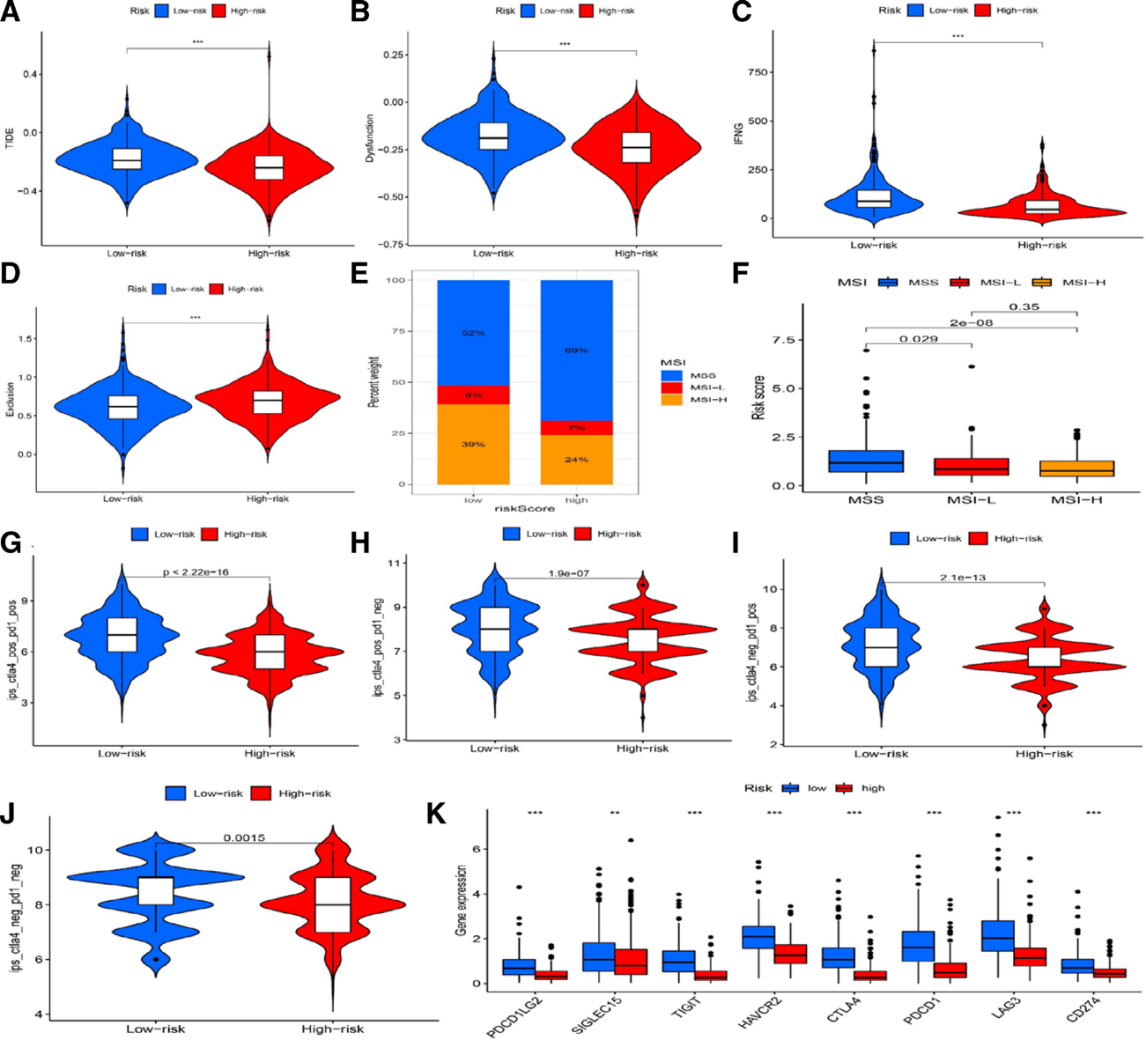
Analysis of immunotherapy response-related genes, IPS, and an ICI in ICD low and high-risk groups. (A–D) Expression of immunotherapy response-related genes revealed by several programs: (A) TIDE, (B) dysfunction, (C) IFNG, and (D) exclusion. (E and F) A comparison of the difference in MSI between the 2 groups. (G–J) A comparison of the difference in IPS between the 2 groups. (K) Differential expression of multiple immune checkpoints between ICD-high and ICD-low subtypes. ICD = immunogenic cell death, ICI = immune-checkpoint inhibitor, IFNG = Interferon Gamma, IPS = immunophenoscore, MSI = Microsatellite instability, TIDE = tumor immune dysfunction score, and exclusion.

The tumor immune dysfunction score, and exclusion score, dysfunction score, and Interferon Gamma score were significantly lower in the high-risk group than in the low-risk group, but the exclusion score was higher (Fig. 9A–D). In the high-risk group, 69% of cases had a microsatellite-stable (MSS) status, and 24% had a high degree of microsatellite instability (MicroSatellite Instability-high [MSI-H]). While in the low-risk group, 52% of cases had a maximum segment size MSS status, and 39% had an MSI-H status (Fig. 9E). As shown in Figure 9F, there was a positive correlation of the risk score with the rate of MSS. Due to excellent efficacy of the immune checkpoint inhibitors (ICI) therapy via blocking the CTLA-4/PD-1 interaction in treatment of some tumors, we also evaluated the role of the ICD score in EC treatment. We utilized the IPS score to assess the potential clinical efficacy of ICIs in different subgroups, showing higher IPS scores in the low-ICD score group, of which the EC patients were more sensitive to immunotherapy than those in the high-ICD-score group (Fig. 9G–J). Since the expression levels of immune-checkpoint genes were closely related to the benefits of immunotherapy, a comparison of the difference in the expression of common immune-checkpoint genes between high- and low-risk groups was also performed. As shown in Figure 9K, 8 checkpoint genes were significantly different between the 2 groups (highly expressed in the low-risk group), including several well-known immunotherapy targets, such as PDCD1 (PD-1), CD274 (Programmed death ligand 1, PD-L1), and CTLA4.

### 3.10. Association of ICD score with chemotherapeutic drugs

**Figure 10.**
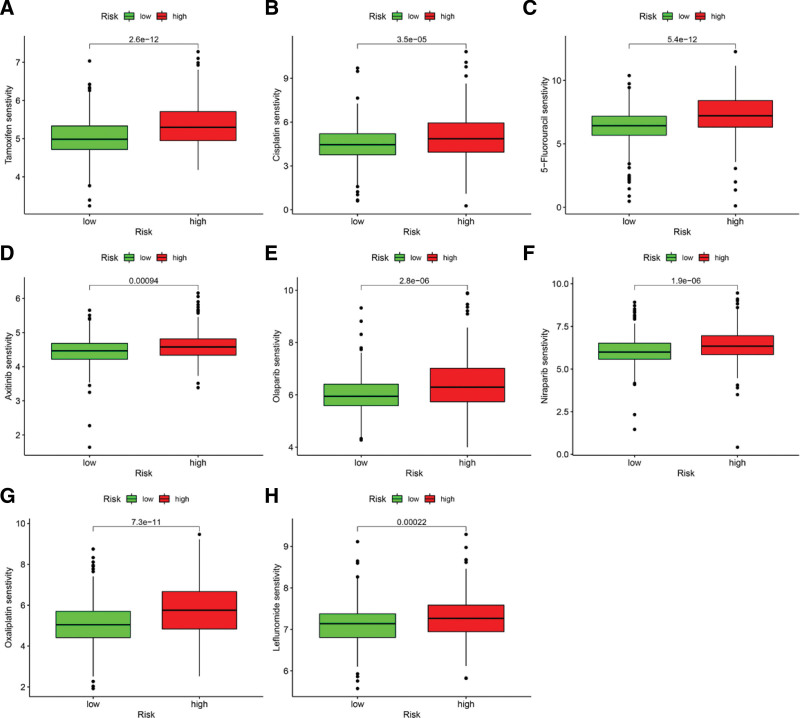
A comparison of treatment efficacy of the following drugs between the high-risk and low-risk groups: (A) tamoxifen, (B) cisplatin, (C) 5-fluorouracil, (D) axitinib, (E) Olaparib, (F) niraparib, (G) oxaliplatin, and (H) leflunomide.

Several chemotherapeutic drugs were recommended for treating EC, including vincristine, doxorubicin, cisplatin, mitomycin, and sorafenib. The treatment efficacy of those drugs between the high-risk and low-risk groups was compared based on the IC50 of those drugs recorded in the TCGA-EC dataset. Compared to the low-risk group, the IC50 values of tamoxifen, cisplatin, 5-fluorouracil, axitinib, olaparib, niraparib, oxaliplatin, and leflunomide were higher in the high-risk group (Fig. 10A–H), suggesting that the EC patients in the low-risk group might be more sensitive to chemotherapeutic drugs.

## 4. Discussion

In this study, we employed bioinformatics analysis to examine the genetic, transcriptional, and clinical data of EC obtained from the TCGA database. The primary objective was to investigate the relationship between ICDRGs and the clinicopathological characteristics of EC patients. The findings of the study indicate that ICD-associated biomarkers can serve as predictors for assessing the immune microenvironment status, chemotherapy susceptibility, and prognosis of EC patients. These results offer valuable insights into the development of personalized treatment strategies for EC patients.

ICD has been described as a unique type of regulated cell death, capable of triggering complete adaptive immunologic responses by DAMPs.^[5,10]^ Significant progress has been achieved in combining immunogenic and novel immunotherapeutic therapies for human cancers,^[14]^ including EC.^[15]^ However, the association of ICD with EC remains elusive. To this end, this study investigated the possible association between ICDRGs and EC via a series of bioinformatic analyses.

First, 2 ICD clusters were identified by consensus clustering based on ICDRGs. According to the Kaplan–Meier survival curve, the cluster ICD high, in which ICDRGs were abundant, had a better prognosis than the cluster ICD low. The efficacy of immunotherapy and the overall survival rate of cancer patients were significantly affected by the complex interaction between tumor cells and the tumor microenvironment.^[16,17]^ In addition, ICD acts as a crucial part of the tumor immune microenvironment.^[18,19]^ Therefore, our study focused on functional enrichment and TME infiltration in the 2 ICD clusters. Functional enrichment analysis revealed that the ICD-high cluster enriched the signaling pathways related to a B cell receptor, T cell receptor, primary immunodeficiency, and chemokine. More immune cell infiltration was observed in the ICD-high cluster, including CD8 T cell and T cells regulatory.

On the other hand, TMB is closely associated with the benefit of immunotherapy in cancer patients.^[20]^ Therefore, our study also compared the differences in somatic mutation distribution between different groups. Phosphatase and tensin homolog, the most frequently mutated gene in copy-number low EC, had a higher coding sequence variants probability in the cluster ICD-high group.^[21,22]^ Since human leukocyte antigen (HLA)-related genes play a crucial role in regulating immune responses,^[23,24]^ our study also included comparing the expression level of HLA-related genes between different groups. Results showed that the expression of most HLA-related genes was upregulated in the cluster-high ICD group. In addition, previous studies revealed that the expression of immune-checkpoint genes is closely associated with the benefits of immunotherapy.^[25–27]^ The expression levels of common immune-checkpoint genes in the 2 cluster subgroups were also compared, which showed a higher expression of the checkpoint genes in the cluster-high ICD group. Notably, several well-known immunotherapy targets, such as PDCD1 (PD-1), CD274 (PD-L1), and CTLA4, were included in the cluster-high ICD group. All the results indicated that the high ICD cluster is associated with immunotherapy responses, which might be a potential biomarker for predicting the response to an ICI.

Next, our study developed an ICD risk signature, which was confirmed to be an independent predictor for the prognosis of EC patients. Compared with the low-risk ICD group, there were significant variations in overall survival, immunization landscape, mutations, TMB, chemotherapy efficacy, and chemotherapeutic drug resistance in the high-risk ICD group. While a low-ICD risk score was significantly associated with a better OS and related to high levels of immune infiltration, lower immune, stromal, and TME scores, suggesting there is more opportunity to trigger an immune response by the tumor in the low-ICD risk group. It has been reported that low TMB is associated with poorer survival.^[28]^ Similar results were also included in our study. Interestingly, we found that ICD scores still affected the survival of EC patients when their TMB score was the same. Besides, most tumor patients with MSI-H had an improved prognosis and a better anti-tumor immune response than those with microSatellite Instability- low (MSI-L) or MSS.^[29]^ Our study observed a higher rate of MSI-H in the low-risk ICD group, which had a better OS and immunotherapy sensitivity than those in the high-risk ICD group, consistent with other reports. Furthermore, there was a negative correlation with the IC50 of tamoxifen, cisplatin, 5-fluorouracil, axitinib, olaparib, niraparib, oxaliplatin, and leflunomide in the low-risk ICD group. We also found higher IPS scores in the low-ICD score group. Consequently, cancer patients in the low-ICD risk group might be the appropriate candidates for receiving the combination of immunotherapy and chemotherapy.

In conclusion, 2 ICD clusters and an ICD risk signature were identified in this study. The association of ICD subtypes with multiple factors was also investigated, such as variations in the immune microenvironment, immunotherapy response, chemotherapy susceptibility, and prognosis for EC. Our findings provide new insights into the individualized treatment strategies for EC patients.

## Acknowledgments

We thank Medjaden Inc. for the scientific editing of this manuscript.

## Author contributions

**Data curation:** Yongjin Luo.

**Project administration:** Xiaoxia Hu.

**Supervision:** Xiaoxia Hu.

**Writing – original draft:** Zhen Liu, Linhong Su.
